# Dacron Graft Intussusception Technique for Treatment of Type A Aortic Dissections: Technical Notes and Preliminary Results

**DOI:** 10.5935/1678-9741.20160042

**Published:** 2016

**Authors:** Bruno Botelho Pinheiro, Walter V. Fagundes, Luís F. F. Muniz, Mats Dreifaldt, Mikael Arbeus, Domingos S. R. Souza

**Affiliations:** 1 Serviço de Cirurgia Cardiovascular Clinicord, Goiânia, GO, Brazil.; 2 Hospital Evangélico Goiano (HEG), Anápolis, GO, Brazil.; 3 Orebro University Hospital, Orebro, Sweden.

**Keywords:** Aortic Diseases, Aorta, Thoracic, Hemorrhage, Cardiovascular Surgical Procedures

## Abstract

**Introduction:**

Optimal surgical management for acute type A aortic dissection (AAAD) remains unclear. The in-hospital mortality rate is still high (15%), and the intraoperative bleeding is an independent risk factor for hospital mortality.

**Objective:**

The aim of our study was describe a new method for aortic anastomosis in the repair of AAAD and report the hospital mortality and bleeding complications.

**Methods:**

Between January 2008 and November 2014, 24 patients, 16 male, median age 62 years, underwent surgical treatment of AAAD. The surgical technique consisted of intussusception of a Dacron tube in the dissected aorta, which is anastomosed with a first line of 2-0 polyester everting mattress suture and a second line of 3-0 polypropylene running suture placed at the outermost side. Open distal anastomosis was performed with bilateral selective antegrade cerebral perfusion in 13 (54.1%) patients.

**Results:**

Cardiopulmonary bypass and aortic clamping time ranged from 75 to 135 min (mean=85 min) and 60 to 100 min (mean=67 min), respectively. The systemic circulatory arrest ranged from 29 to 60 min (mean=44.5 min). One (4.1%) patient required reoperation for bleeding, due to the use of preoperative clopidogrel. The postoperative bleeding was 382-1270 ml (mean=654 ml). We used an average of 4.2 units of red blood cells/patient. There were two (8.3%) hospital deaths, one due to intraoperative bleeding and another due to mesenteric ischemia. The average length of stay in the intensive care unit and hospital was 44 hours and 6.7 days, respectively.

**Conclusion:**

This new method for surgical correction of AAAD was reproducible and resulted in satisfactory clinical outcomes.

**Table:** 

Abbreviations, acronyms & symbols	
AAAD	= Acute type A aortic dissection
ACPB	= Aortic clamping on hypothermic cardiopulmonary bypass
CPB	= Cardiopulmonary bypass
DHCA	= Deep hypothermic circulatory arrest
ICU	= Intensive care unit
IRAD	= International Registry of Acute Aortic Dissections
SD	= Standard deviation
TEE	= Transesophageal echocardiography

## INTRODUCTION

Acute type A aortic dissection (AAAD) is a cardiovascular emergency with a high potential for death. Rapid surgical treatment is indicated to prevent fatal complications. Despite improved surgical techniques and perioperative care, mortality remains high, between 15% and 30%^[1,2]^.

Bleeding from anastomotic sites, especially sites of aorta-to-artificial graft and graft-to-graft anastomoses, is one of the major factors leading to increase in the operative time, rate of blood transfusion and surgical mortality in aortic surgery^[3]^. This type of bleeding may become uncontrollable because of severe coagulopathy, mainly induced by deep hypothermia, a long cardiopulmonar bypass (CPB) time^[4,5]^ or fragile aortic walls by acute aortic dissection^[6]^.

A lot of methods have been reported to prevent this bleeding, such as anastomosis techniques to reinforce a suture line, glues for anastomosis or wrapping methods, such as the Cabrol shunt^[3,6,11]^. However, these techniques are not always perfect and are also complex to be performed for every anastomosis during a tough operation.

The aim of our study was describe a new method for aortic anastomosis in the repair of AAAD and report the hospital mortality and bleeding complications, in three different institutions.

## METHODS

A total of 24 patients from three medical centres (Örebro University Hospital/Sweden; Anis Rassi Hospital/Brazil and Hospital Evangélico Goiano/Brazil) underwent repair of AAAD with a new method for aortic anastomosis between January 2008 and November 2014.

A preoperative diagnosis of aortic dissection was performed by using computed tomography angiography or transesophageal echocardiography (TEE).

Continuous data are presented as means [standard deviation (SD); range]. Categorical data are presented as proportions.

### Technique

Cardiopulmonary bypass was established with venous drainage from the right atrium and return into the axillary or brachiocephalic artery. The systemic temperature was lowered to 32ºC. In patients whose open distal anastomosis was performed the temperature was lowered to 25ºC and bilateral antegrade cerebral perfusion (10 ml/kg/min) was established, via axillary or brachiocephalic artery and left carotid artery. The aorta was cross clamped and opened 1.0 cm above the sinotubular junction.

**Fig. 1:**
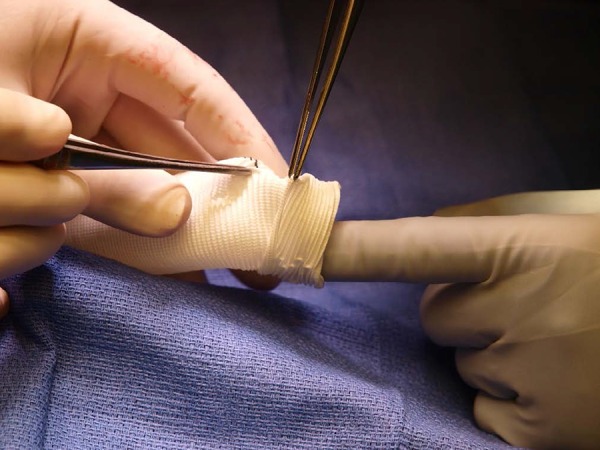
Eversion of the Dacron graft.

**Fig. 2:**
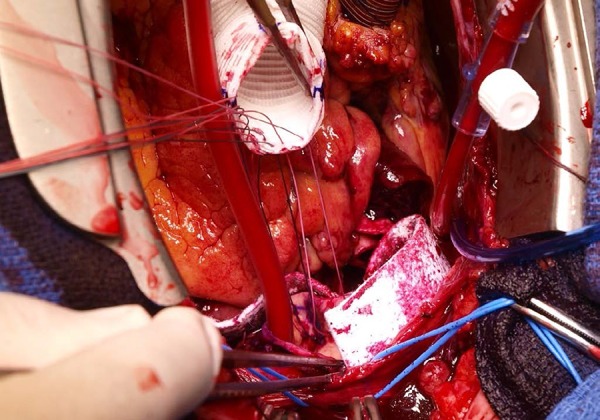
First line of mattress suture passed through an extraluminal circumferential felt strip, the dissected aorta and the graft.

**Fig. 3:**
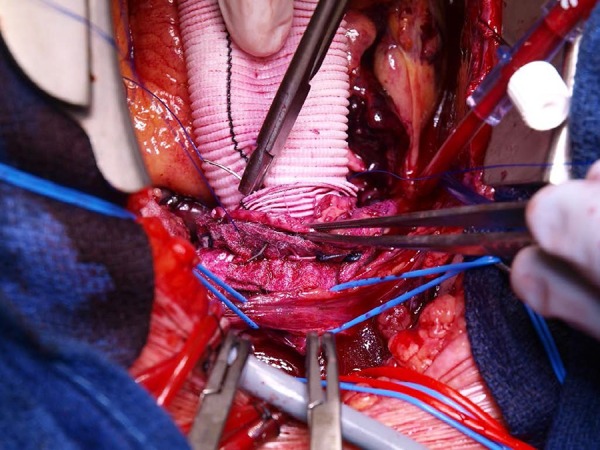
Second line of 3-0 polypropylene running suture.

**Fig. 4:**
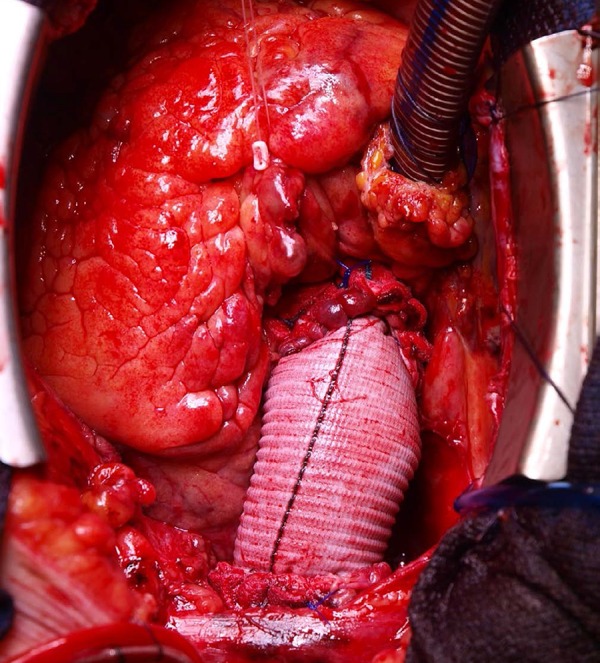
Final aspect.

An appropriate Dacron tube graft, which is usually a little smaller than the diameter of the aortic stump, was selected. The last 3 cm of the end of the graft was everted (Figure 1). Twelve mattress sutures of 2-0 polybutylate-coated braided polyester (Ethibond, Ethicon Inc., Somerville, NJ, USA) was passed through an extraluminal circumferential felt strip, the dissected aorta and the graft (Figure 2). A second line of 3-0 polypropylene (Prolene, Ethicon Inc., Somerville, NJ, USA) running sutures was placed at the outermost side (Figure 3). This technique was used for all distal and proximal anastomoses of the aorta (Figure 4), except for the proximal anastomosis of an aortic root replacement.

Plication of the aortic valve was performed if needed. Substitution of the aortic root with a composite graft (Bentall-DeBono operation) was performed in cases with compromised aortic roots.

## RESULTS

During the study period, 24 consecutive patients underwent surgery for acute ascending aortic dissection. The preoperative data for all patients are shown in Table 1.

**Table 1:** Preoperative patient characteristics (N=24).

Mean age [years (SD; range)]	58 (13; 45-81)
Male/female ratio (%/%)	16/8 (66.6/33.4)
Prior cardiac surgery/PCI	0%/16.6% (N=4)
Hypertension	70.8% (N=17)
Diabetes mellitus	29.1% (N=7)
COPD	20.8% (N=5)
Prior myocardial infarction	8.3% (N=2)
Prior CVA	4.1% (N=1)
Dissection type	
DeBakey type 1	91.7% (N=22)
DeBakey type 2	8.3% (N=2)
Etiology	
Idiopathic	95.8% (N=23)
Marfan	4.1% (N=1)
Systolic LVF	
Good (EF > 50%)	79.2% (N=19)
Impaired (EF < 50%)	20.8% (N=5)
Aortic insufficiency	
None	12.5% (N=3)
Grade I-II	33.3% (N=8)
Grade III-IV	54.2% (N=13)
Shock	25.0% (N=6)
Tamponade	12.5% (N=3)

SD=standard deviation; PCI=percutaneous coronary intervention; COPD=chronic obstructive pulmonary disease; CVA=cerebrovascular accident; LVF=left ventricular function

Cardiopulmonary bypass and aortic clamping time ranged from 75 to 135 min (mean+SD = 85.4±12.7 min) and 60 to 100 min (mean±SD = 67.5±6.8 min), respectively.

In 13 (54.1%) patients, we used an open distal anastomosis at 25ºC with bilateral antegrade cerebral perfusion (10 ml/kg/min). The systemic circulatory arrest ranged from 29 to 60 min (mean±SD = 44.5±4.7 min). One (4.1%) patient presented a postoperative cerebrovascular accident and was discharged with a persistent right hemiplegia.

Bentall-DeBono operation was performed in 10 (41.6%) patients due to a compromised aortic root with severe aortic valve insufficiency. In one (4.1%) patient, additional coronary bypass grafting was performed because of a right coronary ostium disruption due to the dissection.

One (4.1%) patient required reoperation for bleeding, since he was making use of clopidogrel preoperatively. The postoperative bleeding was 382-1270 ml (mean±SD = 654±128 ml). We used an average of 4.2 units of red blood cells/patient (Table 2).

**Table 2:** Perioperative data.

Variable	Mean±SD	Range
CPB (min)	85.4±12.7	75-135
Clamping time (min)	67.5±6.8	60-100
SACP time (min)	44.5±4.7	29-60
Blood transfusion (U)	4.2±1.7	0-12
Blood loss (24h)	654±128	382-1270

CPB=cardiopulmonary bypass; SACP=selective antegrade cerebral perfusion

There were two (8.3%) hospital deaths, one due to intraoperative bleeding (uncontrolled coagulopathy) and another due to mesenteric ischemia. The average length of stay in the Intensive Care Unit (ICU) and hospital was 44 hours and 6.7 days, respectively. Postoperative data are summarized in Table 3.

**Table 3:** Postoperative data.

Variable	n	%
Mortality	2	8.3
Bleeding	1	4.1
Mesenteric ischemia	1	4.1
Morbidity	8	33.3
Reoperation	1	4.1
Cerebrovascular accident	1	4.1
Arrhythmia	4	16.6
Ventilation > 24h	2	8.3
Pnemonia	1	4.1
Renal dysfunction	2	8.3

## DISCUSSION

There is a consensus that AAAD is an urgent surgical disease, given the high mortality in patients who receive medical treatment. The International Registry of Acute Aortic Dissections (IRAD) data have further confirmed this statement: in this registry, which includes aortic centers around the world using different policies regarding diagnosis and management, the overall surgical mortality was 23.9%, whereas patients treated medically had an in-hospital mortality of 58.1%^[1,12]^. Others have also confirmed that surgical results remain suboptimal, showing a mortality rate from 15% to 30%^[1,12]^.

Major complications after surgery for AAAD - bleeding or infection requiring reoperation, renal and respiratory failure, temporary and permanent neurological dysfunction, organ dysfunction, and multisystem organ failure - are worthwhile studying if their prevalence is relatively high and if they substantially influence surgical outcomes, that is, early or late mortality^[13]^.

Persistent oozing and bleeding after aortic anastomosis can occur during aortic surgery. This type of bleeding may become uncontrollable because of severe coagulopathy, mainly induced by deep hypothermia and a long cardiopulmonary bypass time^[4,5]^, or fragile aortic walls by acute aortic dissection^[6]^. A lot of methods have been reported to prevent this bleeding, such as anastomosis techniques to reinforce a suture line, glues for anastomosis or wrapping methods^[3,6,11]^.

With our new surgical technique of intussusception of a Dacron graft in the dissected aorta we had 8.3% hospital mortality (1 intraoperative bleeding; 1 mesenteric ischemia) and 4.1% reoperation for bleeding (one patient in use of clopidogrel).

Chiappini et al.^[14]^ published a large series of 487 consecutive patients with AAAD treated surgically with an in-hospital mortality rate of 22%. Post-operative complications included re-operation for bleeding in 115 (23.8%) patients.

Hansson et al.^[15]^ demonstrated that the 30-day mortality in patients operated for AAAD on dual antiplatelet therapy was 30.4% compared with 13% in patients with no or single antiplatelet therapy (*P*=0.038).

The intraoperative factors of long perfusion time (cardiopulmonary bypass time > 4 hours), prolonged operation time (> 6 hours), and massive blood transfusion (> 20 units) were found to be statistically significant risk factors for hospital death including operative death. Massive blood transfusion during operation can mean technical difficulties, unfavorable anatomy, or intraoperative complications, and these factors can contribute to prolonged operation or cardiopulmonary bypass time and, therefore, affect surgical outcome^[16,18]^.

In our study, cardiopulmonary bypass time ranged from 75 to 135 min (mean=85 min) and the postoperative bleeding was 382-1270 ml (mean=654 ml). We used an average of 4.2 units of red blood cells/patient. None of our patients used > 20 units of blood transfusion.

Stamou et al.^[19]^ compared the early and late clinical outcomes of open distal anastomosis under deep hypothermic circulatory arrest (DHCA) with distal aortic clamping on hypothermic cardiopulmonary bypass (ACPB) during a repair of AAAD. Early postoperative complications, such as cerebrovascular accident and renal failure were comparable between the two groups. A comparable five-year actuarial survival was also observed with both techniques^[19]^. We performed an open distal anastomosis with bilateral antegrade cerebral perfusion in 13 (54.1%) patients, and one patient presented a postoperative cerebrovascular accident in the early postoperative period.

The substitution of the aortic root with a composite graft (Bentall-DeBono operation) was performed in 41.6% of our patients. Bekkers et al.^[20]^. reported long-term results and incidence of reoperations after surgery for AAAD. Late survival was comparable for patients with preserved native valves versus patients with various types of valve replacement. Aortic valve preservation in patients presenting with severe aortic insufficiency was associated with an increased risk of aortic valve reoperation.

### Study Limitations

The main limitation of our study is because it is a nonrandomized trial, with a small sample size. Investigation of the causes of late mortality, late reoperations on the remaining dissected aorta (aortic arch, descending aorta), as well as the fate of the false lumen were beyond the scope of our study and need to be the focus of future reports.

## CONCLUSION

This new surgical technique of intussusception of a graft for surgical correction of AAAD was feasible, reproducible and resulted in satisfactory early outcomes. Future investigations with a larger number of patient, late follow up and randomized methodology may confirm our findings.

**Table:** 

Authors' roles & responsibilities	
BBP	Conception and design study; realization of operations and/or trials; analysis and/or data interpretation; manuscript writing or critical review of its content; final manuscript approval
WVF	Realization of operations and/or trials; approval final manuscript
LFFM	Realization of operations and/or trials; approval final manuscript
MD	Realization of operations and/or trials; approval final manuscript
MA	Realization of operations and/or trials; approval final manuscript
DSRS	Conception and design study; realization of operations and/or trials; analysis and/or data interpretation; final manuscript approval
